# Microtubule architecture *in vitro* and in cells revealed by cryo-electron tomography

**DOI:** 10.1107/S2059798318001948

**Published:** 2018-04-11

**Authors:** Joseph Atherton, Melissa Stouffer, Fiona Francis, Carolyn A. Moores

**Affiliations:** a Institute of Structural and Molecular Biology, Birkbeck College, Malet Street, London WC1E 7HX, England; b INSERM UMR-S 839, 17 Rue du Fer à Moulin, 75005 Paris, France; c Sorbonne Universités, Université Pierre et Marie Curie, 4 Place Jussieu, 75005 Paris, France; d Institut du Fer à Moulin, 17 Rue du Fer à Moulin, 75005 Paris, France

**Keywords:** microtubules, cryo-electron tomography, three-dimensional reconstruction, lattice defects, neurons

## Abstract

The microtubule cytoskeleton is involved in many vital cellular processes. Microtubules act as tracks for molecular motors, and their polymerization and depolymerization can be harnessed to generate force. The structures of microtubules provide key information about the mechanisms by which their cellular roles are accomplished and the physiological context in which these roles are performed. Cryo-electron microscopy allows the visualization of *in vitro*-polymerized microtubules and has provided important insights into their overall morphology and the influence of a range of factors on their structure and dynamics. Cryo-electron tomography can be used to determine the unique three-dimensional structure of individual microtubules and their ends. Here, a previous cryo-electron tomography study of *in vitro*-polymerized GMPCPP-stabilized microtubules is revisited, the findings are compared with new tomograms of dynamic *in vitro* and cellular microtubules, and the information that can be extracted from such data is highlighted. The analysis shows the surprising structural heterogeneity of *in vitro*-polymerized microtubules. Lattice defects can be observed both *in vitro* and in cells. The shared ultrastructural properties in these different populations emphasize the relevance of three-dimensional structures of *in vitro* microtubules for understanding microtubule cellular functions.

## Abstract 2

Electron microscopy is a key methodology for studying microtubule structure and organization. Here, the results of cryo-electron tomography experiments on *in vitro*-polymerized microtubules and comparisons with microtubule ultrastructure in cells are described.

## 1. Introduction   

The microtubule (MT) cytoskeleton is vital for many aspects of cell function, including division, migration and definition of cell architecture. MTs act as tracks for the cellular transport motors kinesin and dynein, but are also dynamic polymers, and both growing and shrinking MT ends are put to work in numerous contexts (Akhmanova & Steinmetz, 2015 ▸; McIntosh *et al.*, 2012 ▸). MTs are built from αβ-tubulin heterodimers that polymerize longitudinally to form polar protofilaments (PFs) and laterally to form the walls of the hollow MTs (Nogales *et al.*, 1998 ▸, 1999 ▸). β-Tubulin is exposed at the so-called plus ends of MTs, which are typically more dynamic, while α-tubulin is exposed at the minus ends. MT dynamics are driven by the tubulin GTPase cycle: GTP-bound tubulin favours MT nucleation and growth, but polymerization stimulates tubulin GTPase activity, yielding MTs built from GDP-bound tubulin which are intrinsically unstable (Desai & Mitchison, 1997 ▸; Mitchison & Kirschner, 1984 ▸).

Electron microscopy (EM) has been a critical tool for studying the polymers, including MTs, and oligomers formed by tubulin *in vitro* since their earliest characterization (Erickson, 1974 ▸). Once cryo-EM had been developed as a near-physiological sample-preparation method (Adrian *et al.*, 1984 ▸), cryo-EM images of *in vitro*-polymerized MTs yielded key insights into the properties of these polymers. These included the range of protofilament (PF) architectures formed *in vitro* (reviewed by Wade & Chrétien, 1993 ▸) and the effect on these architectures of different experimental conditions including nucleotides (Hyman *et al.*, 1992 ▸, 1995 ▸; Ray *et al.*, 1993 ▸; Vale *et al.*, 1994 ▸). Cryo-EM images also showed two-dimensional views of dynamic MT ends *in vitro* (Chrétien *et al.*, 1995 ▸; Mandelkow *et al.*, 1991 ▸), shedding light on the structural basis of MT dynamic instability and highlighting potential mechanisms for its coupling to cellular function (McIntosh *et al.*, 2010 ▸). The application of computational averaging procedures to sets of two-dimensional images of MTs has allowed the determination of their three-dimensional molecular structure, MT binding partners and MT assemblies at increasingly high resolutions (Alushin *et al.*, 2014 ▸; Ichikawa *et al.*, 2017 ▸; Kellogg *et al.*, 2017 ▸; Li *et al.*, 2002 ▸; Nogales *et al.*, 1999 ▸; Nogales & Zhang, 2016 ▸; Vemu *et al.*, 2016 ▸, 2017 ▸; Zhang *et al.*, 2015 ▸).

MTs in a range of physiological contexts in cells and tissues have also been characterized, facilitated by their distinctive architecture (Tilney *et al.*, 1973 ▸) and the fact that MTs are the largest of the cytoskeletal filaments. Electron tomography of resin-embedded, stained samples has provided crucial information about the organization of MT arrays in cells (Redemann *et al.*, 2017 ▸; Stepanek & Pigino, 2016 ▸; Winey *et al.*, 1995 ▸), as well as how MT ends engage with other cellular components (McIntosh *et al.*, 2008 ▸; Moritz *et al.*, 2000 ▸). Cryo-electron tomography, together with subtomogram averaging, has yielded important insight into the organization of MTs and the many other components that form axonemes in eukaryotic flagella at increasingly high resolutions (see, for example, Lin *et al.*, 2014 ▸; Maheshwari *et al.*, 2015 ▸; Oda *et al.*, 2014 ▸). The current quality of microscope and imaging hardware is such that MTs can now be explored in greater detail in three dimensions, *in vitro* and in cells by cryo-EM. This is particularly important for studying MT dynamics because an intrinsic aspect of MT dynamic instability is that the behaviours of individual MTs in a population are uncoupled from each other (Mitchison & Kirschner, 1984 ▸). The resulting potential heterogeneity of MT ends in particular makes averaging problematic. Characterizing MT structure and diversity in three dimensions is important for understanding the molecular basis of MT dynamics and how it is coupled to cellular processes (see, for example, Guesdon *et al.*, 2016 ▸).

We recently explored the structures of MT ends *in vitro* in the context of our work on the mechanism of MT minus-end binding by the CAMSAP family of proteins (Atherton *et al.*, 2017 ▸). CAMSAP proteins recognize the conformations of polymerized tubulin specific to MT minus ends (Atherton *et al.*, 2017 ▸), are important for the stabilization of non­centrosomal MTs (Jiang *et al.*, 2014 ▸; Hendershott & Vale, 2014 ▸) and contribute to tissue architecture and differentiation (Chuang *et al.*, 2014 ▸; Noordstra *et al.*, 2016 ▸; Tanaka *et al.*, 2012 ▸; Toya *et al.*, 2016 ▸; Yau *et al.*, 2014 ▸). To understand how these proteins specifically target and protect MT minus ends, we used cryo-EM imaging to visualize the ends of *in vitro*-polymerized stable GMPCPP MTs in three dimensions. Here, we detail our approach to these experiments, the analysis of the data and their interpretation. We also compare our *in vitro* findings concerning stable GMPCPP MTs with observations of dynamic MTs *in vitro* and in cultured neurons (Leterrier *et al.*, 2017 ▸). The cellular environment presents additional challenges to structural characterization of the MT cytoskeleton, but provides an informative backdrop with which to begin to evaluate the architecture of MTs in their functional context.

## 2. Studying MT ultrastructure *in vitro*   

### 2.1. Sample preparation of stable MTs *in vitro* for cryo-electron tomography data collection   

As described previously (Atherton *et al.*, 2017 ▸), double-cycled bovine brain tubulin GMPCPP MTs (Cytoskeleton) were polymerized at a final concentration of 5 mg ml^−1^ in BRB80 (80 m*M* PIPES, 2 m*M* MgCl_2_, 1 m*M* EGTA, 2 m*M* DTT pH 6.8) with 2 m*M* GMPCPP for 30 min at 37°C. The MTs were pelleted at room temperature, the supernatant was removed and the MTs were depolymerized by resuspension in BRB80 at 4°C and incubation on ice for 5 min. GMPCPP was then added to 2 m*M* and the MTs were repolymerized by incubation at 37°C for 45 min. These GMPCPP MTs were centrifuged and resuspended in BRB80 at 30°C. C-flat holey carbon grids (Protochips) with 2 µm holes were glow-discharged in air and 4 µl of 10 nm nanogold fiducial–BSA solution (Sigma) was added and then blotted prior to the addition of MTs. 4 µl of MTs at 0.25 mg ml^−1^ were applied to grids, incubated for 30 s in a Vitrobot (FEI) operating at 30°C and 100% humidity, blotted from both sides and vitrified in liquid ethane.

Single-axis tilt series at defoci between ∼−3 and −5 µm were collected using a Tecnai G2 Polara at 300 kV with a Quantum post-column energy filter (Gatan) operated in zero-loss imaging mode with a 20 eV energy-selecting slit as described previously (Atherton *et al.*, 2017 ▸). Movies were recorded on a K2 Summit direct electron detector (Gatan) operating in counting mode, with a final sampling of 5.39 Å per pixel at 5 e^−^ per pixel per second. MTs are visible in the search mode of the low-dose setup. Together with consideration of ice thickness (thick ice renders MTs hard to see, while very thin ice makes the MTs visibly squashed), this allowed suitable areas for tilt-series data collection to be identified.

Tilt series of 60 or 80 e^−^ Å^−2^ total dose were collected in 3° increments in a bidirectional manner to maxima of −60 and 60°, with higher tilts receiving compensatory higher electron doses. For drift correction at each tilt angle, subframes (four frames per second) were aligned during collection using *DigitalMicrograph* for calculation of the final tilt-series stack.

### 2.2. Cryo-electron tomography image processing and analysis of *in vitro*-polymerized MTs   

Unbinned tilt series were processed and tomograms were generated using the Etomo graphical user interface to *IMOD* (v.4.7.15). 10 nm gold fiducials, selected manually based on minimal observed beam-induced movement, were used for tilt-series alignment. CTF correction was not performed on these data. The final tomograms were binned by three to reduce noise. Following three-dimensional reconstruction, MT ends were further characterized using the 3*DMOD* ‘slicer’ viewer. The trajectories of individual PFs were manually traced by determining their centres of mass in successive sections along the MT axis. These PF coordinates were also used to evaluate the relative positions of PFs within each MT. To aid moiré-pattern visualization and thus PF number determination, rectangular masks were applied around the origin of Fourier transforms of individual MTs and were back-transformed using *FIJI* (Schindelin *et al.*, 2012 ▸). Determination of the PF number in tomograms was performed by extracting transverse sections of MTs in 3*DMOD*, centring these images and then applying different rotational symmetries in *IMAGIC* (Guesdon *et al.*, 2016 ▸; van Heel *et al.*, 1996 ▸; von Loeffelholz *et al.*, 2017 ▸). Automated segmentation of 3× binned cryo-tomograms was performed using the *tomoseg* module of *EMAN*2 v.2.2 (Chen *et al.*, 2017 ▸).

### 2.3. Ultrastructure of stable MTs *in vitro*   

**Figure 1:**
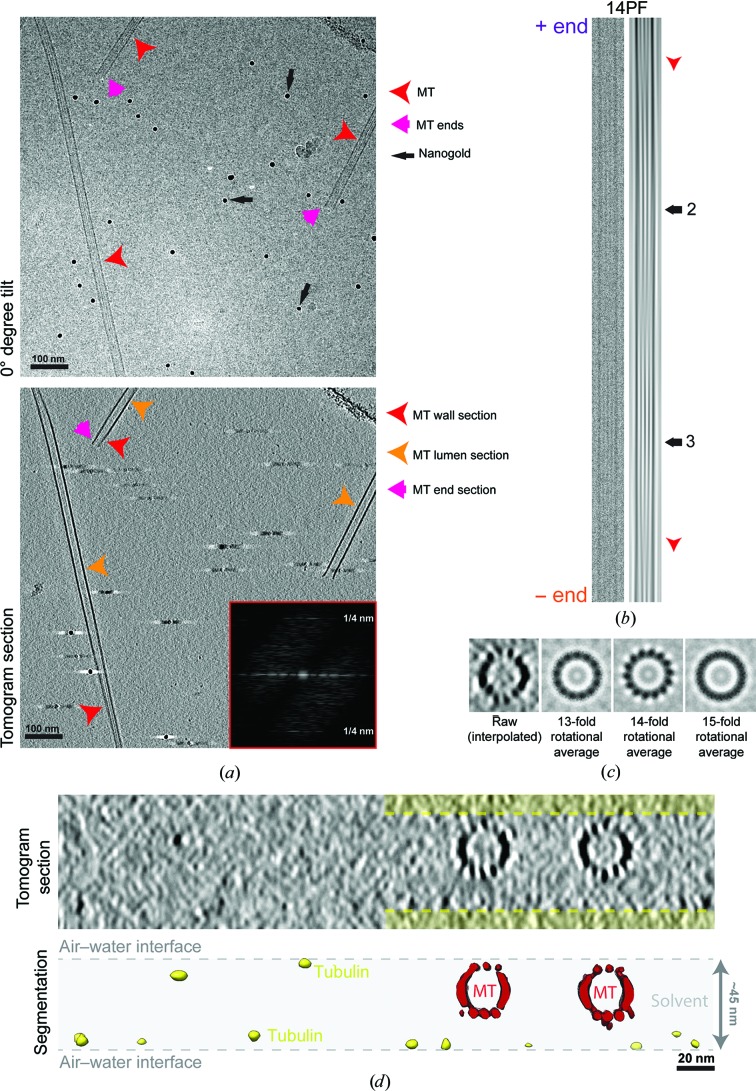
GMPCPP-stabilized *in vitro*-polymerized MTs visualized by cryo-electron tomography. (*a*) Top, 0° tilt image (motion-corrected movie sum) from a tilt series, showing a typical field of MTs that includes views of the MT lattice (red arrowhead, including the moiré pattern) and MT ends (pink arrow), together with 10 nm nanogold fiducial markers (black arrows). Bottom, 8 nm section through the three-dimensional tomographic reconstruction of the same sample, showing the appearance of sections through the MT wall (red arrowhead) in which individual PFs are visible, the MT lumen (orange arrowhead) and MT ends (pink arrow). Inset: Fourier transform of an extracted MT showing a layer line corresponding to the 4 nm spacing of the tubulin monomers within the MT lattice, indicative of the level of structural detail in these data. (*b*) An exemplar MT image (0° tilt image; left) and Fourier filtered image (right) showing the moiré pattern characteristic of a 14PF MT (black arrows). The arrowhead shapes within the moiré pattern (red arrowheads) indicate the MT polarity, with the MT plus end towards the top in this case. (*c*) Rotational averaging analysis of 8 nm thick transverse sections from the tomographic reconstruction supports the moiré-pattern-based assignment of 14PF architecture for this MT, yielding clear PF projections in the 14-­fold average, while 13-fold or 15-fold averaging of the same data blurs out structural information from the characteristic PF projection. (*d*) Transverse section (top) and segmented and surface-rendered view (bottom) through the same three-dimensional tomogram, showing the distribution of the MTs (red) in the vitreous ice layer (indicated by the shaded area) and the tendency of the background protein (tubulin, yellow) to lie at the air–water interface of the sample. This view also shows the distorting effect of the missing wedge on the MT structure, illustrating the difficulty of directly counting PFs in such data.

As part of our study of MT minus-end recognition by CAMSAP family members, we used cryo-ET to study the three-dimensional structure of GMPCPP-stabilized MTs polymerized *in vitro*. Stable GMPCPP minus ends are good substrates for CKK binding (Jiang *et al.*, 2014 ▸; Hendershott & Vale, 2014 ▸; Atherton *et al.*, 2017 ▸), emphasizing the functional relevance of the three-dimensional organization of these MTs. Furthermore, the ends of these MTs are homogenous with respect to their dynamic state (*i.e.* they are all stable), as opposed to dynamic MTs where individual MTs may be growing, shrinking or pausing as dictated by MT dynamic instability (see below). From a practical perspective, because these MTs are less sensitive to temperature or dilution, sample preparation for cryo-EM is straightforward. Their stability also means that the protein background is very low in cryo-ET tilt-series images, reducing the noise in the tomographic reconstructions and allowing detailed insight into the ultrastructure of these MTs and their ends, including information about the 4 nm tubulin repeat within the MT PFs (Fig. 1 ▸).

Two-dimensional projections of cryopreserved MTs reveal moiré patterns that arise from superposition of the PFs in the three-dimensional MT wall and provide information about the underlying MT architecture (Chrétien & Wade, 1991 ▸; Wade *et al.*, 1990 ▸). Correlations between moiré-pattern repeat length, underlying PF number and PF orientation with respect to the MT long axis have been described showing that, according to the PF number, straight or skewed PFs can be accommodated by the MT lattice. GMPCPP favours the polymerization of predominantly 14PF MTs (Hyman *et al.*, 1995 ▸; Vale *et al.*, 1994 ▸), consistent with the ∼500 nm moiré repeat observed in our MTs, which we observe in the 0° tilt image despite the very low dose (<2 e^−^) used (Figs. 1 ▸ *a* and 1 ▸ *b*).

The MT moiré-pattern repeat is a useful metric even when collecting cryo-ET data for three-dimensional reconstruction (Fig. 1 ▸ *b*): the impact of the missing wedge on tomographic reconstructions, especially in single-tilt data collection, is such that it is very hard to accurately count the PF number directly in three-dimensional tomographic reconstructions (Fig. 1 ▸ *c*). However, two-dimensional analysis of sequential transverse sections through the three-dimensional tomogram can be used to validate PF number assignment, and is particularly useful for very short MT segments that are not long enough to show a complete moiré pattern (Guesdon *et al.*, 2016 ▸; von Loeffelholz *et al.*, 2017 ▸; Fig. 1 ▸ *c*; see also Figs. 3*a* and 4*c* below).

The three-dimensional tomograms can also be used to visualize where the MTs lie in the layer of vitreous ice in which they are imaged (Fig. 1 ▸ *d*), reflecting their location after blotting and at the moment of vitrification. Typically, the MTs sit within the ice layer, although sometimes one surface is in contact with the air–water interface. This presumably reflects in part that the selection of intact-seeming MTs, rather than those clearly deformed by the thinness of the solvent layer, is part of the manual screening process for areas from which to collect tilt series. This view also shows the very low background found in these stabilized MT samples, although interestingly what background there is (presumably tubulin) tends to be located towards the air–water interface.

As described previously (Chrétien *et al.*, 1996 ▸; Atherton *et al.*, 2017 ▸), the moiré pattern can also be used to determine MT polarity by (i) tracking the direction of the moiré-pattern ‘arrowhead’ in two-dimensional projections and (ii) tracking the change in the appearance of the moiré pattern with tilt angle in each tilt series, which is more reliable. Because of the PF skew intrinsic to their architecture, GMPCPP 14PF MTs can be analysed in this way (Fig. 1 ▸ *b*), further emphasizing their usefulness in the differentiation of plus/minus ends for understanding CAMSAP binding. 13PF MTs are built from practically straight PFs, making polarity assignment challenging.

**Figure 2:**
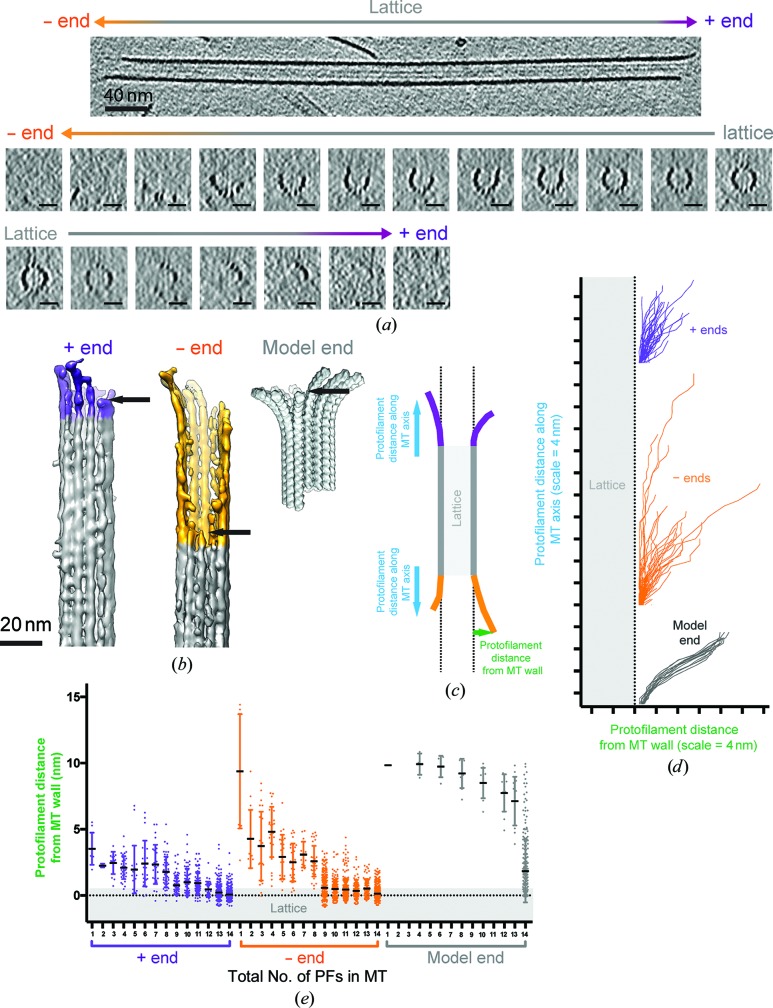
GMPCPP MT end structure visualized by cryo-electron tomography. (*a*) Top, longitudinal slice through the tomographic reconstruction, showing an MT in which both plus and minus ends (as indicated and assigned using the moiré pattern) are visible. Bottom, a series of ∼5 nm transverse sections through the tomographic reconstruction showing the transition from lattice to end. At both ends the cylindrical organization of the MT is retained even as PFs are lost, until a critical point (see below) when the PFs curve gently away from the MT axis in a sheet, often remaining laterally connected until the very end of the MT. (*b*) Segmented and three-dimensional surface-rendered volumes of a plus (purple) and minus (yellow) MT end and a model minus-end volume constructed from straight (PDB entry 3jat; Zhang *et al.*, 2015 ▸) and curved (PDB entry 3ryh; Nawrotek *et al.*, 2011 ▸) tubulin conformations; the arrow indicates the beginning of tapering along the MT axis in each case. PDB entry 3ryh at 9.5° curvature was selected because it is a curved GTP-bound structure of four monomers in length that is useful for model building, but numerous structures of curved tubulin have been determined that show a range of curvatures (∼9.5–13°; for a review and calculation of curvature, see Brouhard & Rice, 2016 ▸). (*c*) Schematic representation of the PF position at MT ends relative to the MT wall. (*d*) Graphical representation from subtomograms of five plus (purple) and five minus (yellow) ends, plotting three-dimensional PF trajectories as they transition from MT lattice to MT end. These plots show that while there is a range of PF curvature and length at MT ends, PFs at plus and minus ends are not as curved as a PDB-based model PF; for the plus end *n* = 62 PFs and for the minus end *n* = 63 PFs. (*e*) Graphical representation from five subtomogram(s) of the loss of PFs from MT ends showing that at both ends, after approximately five PFs are lost from the 14PF architecture, the remaining PFs abruptly start curving outward as an MT sheet, *i.e.* in our data GMPCPP MTs need at least ∼9 PFs to maintain their cylindrical structure. The model end (grey) is built such that curved and lattice-constrained PFs superimpose on each other, leading to the data spread in the plot. In each case, whisker plots show the mean ± standard deviation, with individual measurements for all PFs shown as a scatter plot.

The ability to accurately determine the polarity of GMPCPP 14PF MTs allowed us to characterize the ultrastructure of plus and minus ends in these stable MTs (Atherton *et al.*, 2017 ▸; Fig. 2 ▸). Visualization of polymer ends also emphasizes that the definition of what constitutes an MT end is rather ambiguous. Our cryo-ET reconstructions show that MTs start to lose PFs while retaining their overall cylindrical architecture (Figs. 2 ▸ *a* and 2 ▸ *b*). It is only after a number of PFs have been lost that the cylindrical MT wall opens out to a curved MT sheet. These transitions are found at both MT plus and minus ends. Overall, we found that the three-dimensional structures of MT ends are highly variable, showing a range of PF tapers and lengths, even when only plus ends and only minus ends are compared with each other. This variability is illustrated as a PF plot (Figs. 2 ▸ *c* and 2 ▸ *d*) and is compared with a model 14PF MT end prepared using exemplar structures of curved tubulin found in the PDB (Fig. 2 ▸ *b*). These model PFs have, by design, uniform curvature and do not reflect the influence of lateral PF interactions on curvature. In contrast, overlays of PFs from several plus and minus ends show a wide variation in length and curvature.

When the number of PFs per MT are plotted against their distance from the MT wall (Fig. 2 ▸ *e*), a distinct transition can be observed from a cylindrical MT lattice to an MT sheet which pulls away from the MT wall when the number of PFs is nine or less. This is seen at both the plus and minus ends. The minus-end data set contained more PFs with long extensions both along the MT axis (see also Fig. 2 ▸ *d*) and pulled away from the MT wall; however, owing to the high variance in the data set these differences between plus and minus ends were not statistically significant (Student’s t-test). Even as the sheet opens out, PF curvature is generally constrained compared with the tubulin curvature typically seen in tubulin crystal structures (Figs. 2 ▸ *b* and 2 ▸ *d*). It is therefore clear that the majority of PFs at stable MT ends adopt a curved state intermediate between the lattice and the curvature seen in crystal structures and closer to that described for cold-induced GMPCPP-stabilized tubulin tubes (Wang & Nogales, 2005 ▸).

Overall, this analysis led us to conclude that MT plus and minus ends have similar ultrastructures when judged at this resolution, and therefore that global differences in ultrastructure are un­likely to be the basis of CAMSAP minus-end recognition. Using single-particle cryo-EM reconstruction, we found that the fragment of CAMSAP that defines MT minus-end binding specificity, the so-called CKK domain, binds between MT PFs. Furthermore, a mutant CKK domain with reduced minus-end binding specificity bound at the same site as the wild-type CKK but with a subtly altered orientation with respect to the MT. High-resolution TIRF microscopy also revealed that CAMSAPs are insensitive to the MT nucleotide state. They do not bind at the extreme MT minus end, but rather bind at a position equivalent to the curved, sheet-like region that we characterized by cryo-ET. We thus concluded that the CAMSAP minus-end recognition mechanism depends on subtle changes in tubulin curvature in the transition between MT lattice and curved tubulin sheet that is predicted to be different between plus and minus MT ends (Atherton *et al.*, 2017 ▸).

**Figure 3:**
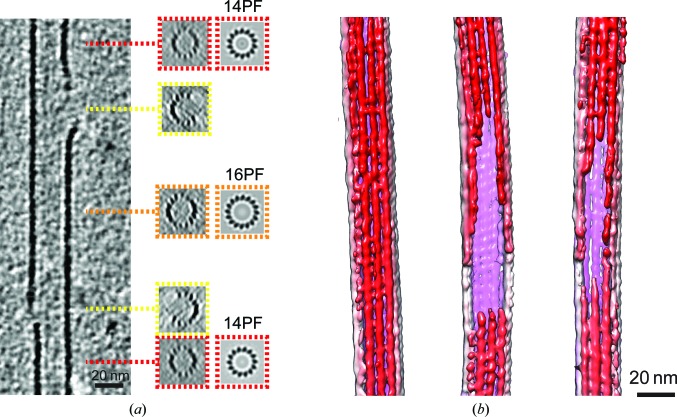
Cryo-electron tomography of GMPCPP MTs reveals lattice defects. (*a*) Longitudinal section through a three-dimensional tomographic reconstruction in which two different lattice defects are visible. Insets, transverse sections through the same MT with the position of the section relative to the polymer and their rotational average indicated in colour. These show the PF mismatches at the defects. (*b*) Three-dimensional rendering of examples of MTs with the near-surface MT coloured red and more distant MT surfaces coloured pink; left, an MT without a defect; middle and right, different MTs with lattice defects.

Strikingly, our tomograms also reveal lattice defects in *in vitro*-polymerized GMPCPP MTs (Fig. 3 ▸), where we define defects as breaks in an otherwise complete MT lattice that involve temporary loss of one or a subset of PFs. The distorting effect of the missing wedge makes it hard to measure the frequency of such defects precisely. However, in our sample we confidently identified 19 defects of various types and sizes in 25 MTs (one defect per 1250 nm of MT polymer). These could have arisen by a number of mechanisms, for example MT end-to-end annealing in solution prior to cryo-EM sample preparation (Chrétien *et al.*, 1992 ▸), physical damage during cryo-EM sample preparation or incorporation of lattice defects during MT polymerization *in vitro*. For example, some lattice defects were observed to delineate sections of MTs with different PF numbers (Fig. 3 ▸ *a*). Given the MT arrangement in this region of the polymer, this defect type might be expected to form on MT annealing, although 16PF MTs are very rare under the polymerization conditions we used.

In nontomographic cryo-EM data sets collected for three-dimensional reconstruction, MT images are mainly selected by overall PF architecture and often according to the extent of decoration by MT binding partners. If present in MT populations used for single-particle imaging experiments, lattice defects could degrade or distort high-resolution three-dimensional structure deter­mination. In practice, some discontinuities can be spotted and removed during semi-automated particle picking. If they are not extracted manually, they might also be excluded from subsequent reconstruction steps owing to aberrant alignment or two-dimensional classification. If such rare defect-containing data are incorporated into single-particle reconstructions, we predict that they mainly contribute noise.

Lattice defects and their repair have been characterized *in vitro* and in cells and linked to aspects of dynamic instability; in particular, such self-repair events are reported to promote MT rescue (Schaedel *et al.*, 2015 ▸; de Forges *et al.*, 2016 ▸; Aumeier *et al.*, 2016 ▸; for further cryo-ET evidence, see Schaedel *et al.*, 2018 ▸). They may be induced, for example, by the binding of drugs such as eribulin to MTs (Doodhi *et al.*, 2016 ▸). This emphasizes the interest in these under-reported phenomena and the power of cryo-ET to characterize them.

### 2.4. Sample preparation of dynamic MTs *in vitro* for cryo-electron tomography data collection   

To prepare dynamic GDP/GTP MTs for cryo-electron tomography (cryo-ET), bovine brain tubulin was polymerized at a final concentration of 5 mg ml^−1^ in BRB80 with 1 m*M* GTP for 3 min in a heat block at 37°C. 4 µl was applied to EM grids in a vitrobot (FEI) operating at 37°C and 100% humidity, and MTs were allowed to polymerize on the grid for a further 2 min before blotting on both sides and vitrification in liquid ethane. As described in §[Sec sec2.1]2.1, C-flat holey carbon grids with 2 µm holes were glow-discharged in air and 4 µl of 10 nm nanogold fiducial–BSA solution (Sigma) was added and then blotted prior to the addition of MTs. Data collection and image processing for this sample were the same as for stable MTs (§§[Sec sec2.1]2.1 and [Sec sec2.2]2.2).

### 2.5. Ultrastructure of dynamic MTs *in vitro*   

**Figure 4:**
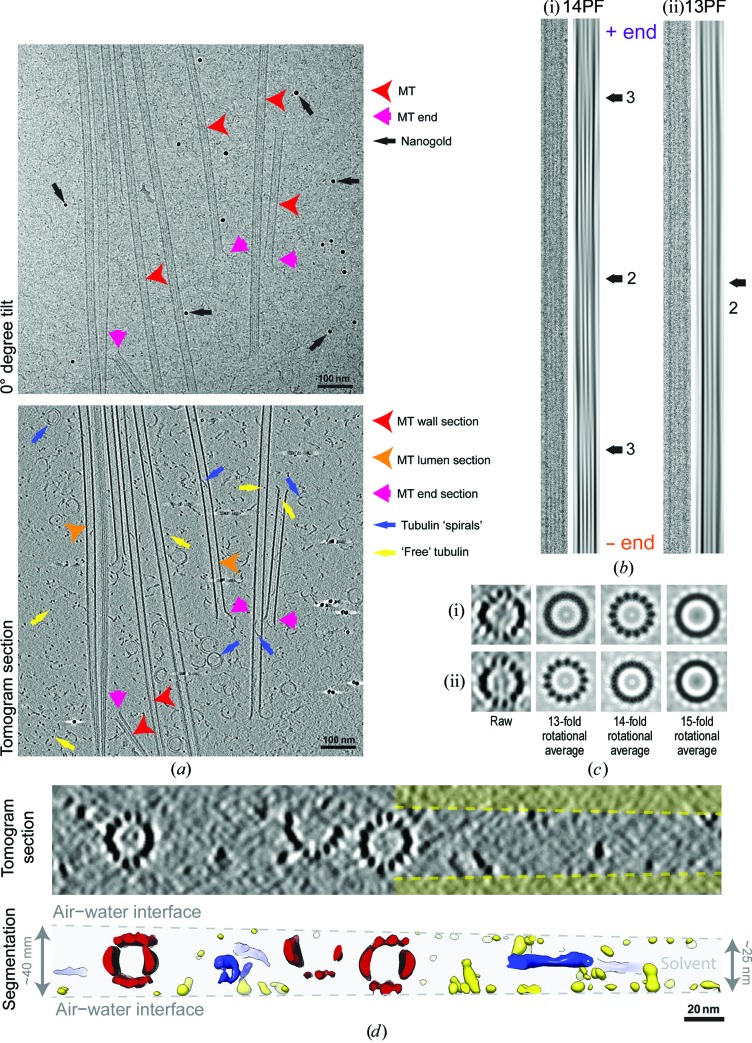
Dynamic *in vitro*-polymerized MTs visualized by cryo-electron tomography. (*a*) Top, 0° tilt image (motion-corrected movie sum; ∼2 e^−^ Å^−2^ total dose) from a tilt series, showing a typical field of MTs that includes views of the MT lattice (red arrowheads, including the moiré pattern) and MT ends (pink arrows), together with 10 nm gold fiducial markers. Bottom, 8 nm section through the three-dimensional tomographic reconstruction of the same sample, showing the appearance of sections through the MT wall (red arrowhead) in which individual PFs are visible, the MT lumen (orange arrowheads), MT ends (pink arrow), tubulin PF spirals (blue arrows) and unpolymerized tubulin (yellow arrows). (*b*) Exemplar 0° tilt MT image (left) and Fourier filtered image (right) showing the moiré patterns characteristic of (i) a 14PF MT and (ii) a 13PF MT. Because the 14PF MT is not sitting perfectly flat within the plane of the vitreous ice, the arrowhead shape within the moiré pattern that might be used to indicate MT polarity is not visible, but its polarity was determined using the change in appearance of the moiré pattern with the tilt angle. Because the PFs lie parallel to the MT axis in the architecture of the 13PF MT, the moiré pattern does not vary along the MT length and its polarity cannot be determined using this approach. (*c*) Rotational averaging analysis of 8 nm thick transverse sections from the tomographic reconstruction supports the moiré-pattern-based assignment of PF architecture for these MTs. (*d*) Transverse section (top) and segmented and surface-rendered view (bottom) through the same tomogram, showing the distribution of the MTs (red) in the vitreous ice layer, the tendency of background protein (tubulin, yellow) to lie more at the air–water interface of the sample and the tendency of the tubulin spirals (blue) to lie horizontally at the centre of the ice layer.

As noted above and in contrast to GMPCPP MTs, a defining property of dynamic instability is that individual dynamic MTs in a population will exhibit dynamics independently of each other. These images therefore show MTs at all states of growth, shrinkage and pause (Fig. 4 ▸ *a*). As implied by the sample-preparation procedure (§[Sec sec2.4]2.4), dynamic MTs require much more careful handling compared with stabilized MTs because they are highly sensitive to both temperature and tubulin concentration. In addition, since MT growth requires free tubulin, and depolymerizing MTs release free tubulin to solution, dynamic MT samples exhibit comparatively high levels of protein in the background. The high protein background in these samples is emphasized by the presence of tubulin dimers and curved tubulin oligomers inside MTs (Fig. 4 ▸ *a*). We speculate that during MT polymerization in these dynamic samples, some soluble tubulin and tubulin oligomers are randomly trapped inside the MT cylinder as the wave of polymerization passes around them just prior to vitrification. In the images of these dynamic MTs, a greater range of moiré patterns and therefore PF architectures are also observable, with the majority of MTs containing 13 or 14 PFs (Fig. 4 ▸ *b*).

The tomograms reveal where the dynamic MTs lie in the cryo-ET ice layer (Fig. 4 ▸ *c*). Completely polymerized MTs vary in position within the ice layer, and the ring-shaped curved tubulin PFs, which are likely to be detached depolymerization products, typically lie flat, giving their distinctive view in two-dimensional projection images. In contrast, the majority of unpolymerized tubulin tends to lie at the air–water interface (shown in yellow in Fig. 4 ▸ *c*), where it may be subjected to potentially denaturing forces and thus will be unavailable for polymerization (Glaeser *et al.*, 1991 ▸). This presents a particular problem when correlating MT dynamic parameters at a range of tubulin concentrations with the appearance of the MT population at the same nominal tubulin concentrations by cryo-EM. It is not known whether the amount of protein found at the air–water interface in a cryo-EM sample is linearly related to the total protein concentration (Noble *et al.*, 2017 ▸). Thus, the tubulin available to incorporate into dynamic MT ends in cryo-EM samples may vary in complex ways compared with the tubulin present in bulk solution. Therefore, the interpretation of the appearance of MT ends with respect to particular parameters of dynamic instability should be approached with extreme caution. Rather, a more conservative approach can be taken, using trends in the appearance of MT ends in cryo-EM samples at different bulk tubulin concentrations to give indications of structural trends in dynamic instability (Vemu *et al.*, 2017 ▸). Future development of improved cryo-EM sample preparation that mitigates against protein migration to the air–water interface will enable this key problem in MT biology to be tackled.

**Figure 5:**
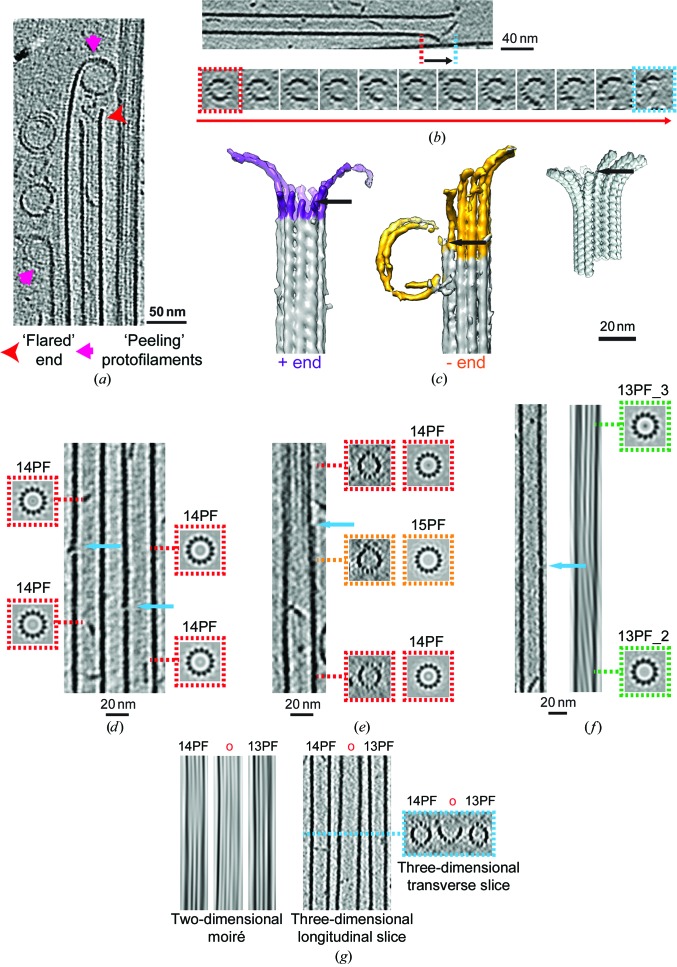
Dynamic MT end structure visualized *in vitro* by cryo-electron tomography. (*a*) Longitudinal slice through the tomographic reconstruction, showing a range of MT end morphologies including the highly characteristic ‘rams horn’-like appearance associated with depolymerizing MTs (pink arrows) and the relatively short flared region associated with growing MTs (red arrowhead). (*b*) Top, longitudinal slice through the tomographic reconstruction, showing a dynamic MT (of unknown polarity) in which one end is visible. Bottom, a series of transverse sections through the tomographic reconstruction, showing the transition from lattice to end. As was seen in GMPCPP MTs (Fig. 2 ▸ *a*), the cylindrical organization of the MT is retained even as PFs are lost, until a critical point when the PFs curve gently away from the MT axis in a sheet, often remaining laterally connected until the very end of the MT. (*c*) Three-dimensional surface rendering of plus (purple, left) and minus (yellow, middle) depolymerizing MT ends compared with a model MT end as described for Fig. 2 ▸(*b*). A range of tubulin curvature is observed in minus and plus ends, with peeling PFs exhibiting a similar curvature to curved tubulin PDB structures. (*d*) Centre, longitudinal section through a tomogram of two MTs each with a small lattice defect indicated by blue arrows. Insets, transverse sections through these MTs with positions of sections relative to the MT and their rotational average indicated in colour. (*e*) Left, longitudinal section through a tomogram of an MT, with a blue arrow indicating a small lattice defect. Insets, transverse sections through the same MT with the position of sections relative to the polymer and their rotational average indicated in colour, showing the change in PF number that coincides with the defect. (*f*) Left, longitudinal section through a tomogram of a 13PF MT, with a blue arrow indicating a small lattice defect that coincides with a change in helical symmetry. Insets; centre, Fourier filtered two-dimensional projection of this MT, showing a change in characteristic moiré pattern from a 13PF with a 3-start (top section) to a 13PF with a 2-start (bottom section); right, transverse sections through the same MT with the position of the section relative to the polymer and their rotational average indicated in colour. (*g*) Left, Fourier filtered two-dimensional projection image of three MTs: a 14PF (left), a 13PF (right) and an incomplete tube (centre, red ‘o’ for ‘open’). Centre, longitudinal slice through a three-dimensional tomogram of the three MTs. Right; transverse slice, at the position indicated with a light blue dashed line, through the three-dimensional tomogram of the three MTs. Note the less complex moiré pattern of the incomplete tube because only a single polymer surface is projected in the image.

The tomograms of these dynamic MTs show the highly heterogeneous three-dimensional structure of tubulin at all phases of MT dynamic instability (Fig. 5 ▸ *a*). Even with the higher protein background in this sample owing to the free tubulin, considerable structural detail is available in these samples. A subset of (presumably growing) MTs exhibit the same lattice-to-end ultrastructural transitions (Fig. 5 ▸ *b*) as seen in the GMPCPP-stabilized ends (Fig. 2 ▸ *a*), in which the number of PFs in the cylindrical MT wall decreases towards the MT end until the point where the cylinder opens out into a short, sheet-like structure. The three-dimensional organization of depolymerizing plus and minus ends are similarly complex, being composed of differently curving PFs. The protruding PFs that curl away from the ends exhibit a similar high curvature to that seen in tubulin crystal structures, as illustrated in the model of a MT end built from these available crystal structures (Fig. 5 ▸ *c*).

A number of lattice defects were also seen in dynamic MT preparations. The most common were small discontinuities in the lattice apparently less than the size of a tubulin dimer (Fig. 5 ▸ *d*). These presumably arise from imperfect preservation of the ordered lattice array during polymerization, resulting in a small curved PF protrusion from the MT wall. In most defects found in dynamic MTs, the same PF number is retained on either side. Some examples were found, however, where a change in PF number (Fig. 5 ▸ *e*) or a change in helical symmetry with the same number of PFs (Fig. 5 ▸ *f*) were observed either side of these lattice defects. Larger breaks in the GDP lattice were also observed in the form of lengths of incomplete MTs in many cases, resulting in a flatter lateral curvature in the remaining half tube (Fig. 5 ▸ *g*). In the dynamic MT preparation, such defects were often observed at physical barriers such as MT crossing points or where MTs encounter the edges of carbon holes. Since dynamic MTs are grown at least partly on the grid, the polymers are handled less prior to vitirification than the GMPCPP MTs. Furthermore, the maximum lifetime of these MTs prior to vitrification is 5 min (compared with hours for the GMPCPP MTs), leaving little time for annealing with other MTs. Therefore, in our dynamic MT samples, defects are both more likely to be observed during nucleation or polymerization and are less likely to result from annealing or mechanical handling forces.

Overall, cryo-EM studies of *in vitro*-polymerized MTs reveal the heterogeneity of the resulting architectures, reflecting the potential plasticity of inter-subunit contacts within the MT lattice.

## 3. Ultrastructure of dynamic MT ends in cells   

### 3.1. Culture of neurons and preparation for cryo-EM   

QUANTIFOIL R 2/4 gold G200F1 Finder EM grids were added to the surface of plastic culture dishes and sterilized under UV light. The EM-grid and dish surfaces were then coated with poly-d-lysine (Millipore) and mouse laminin (Thermofisher). Hippocampal primary cultures were isolated from E17.5 mouse embryos on an Sv-129 background. Tissue was triturated after trypsin/DNaseI (both from Sigma) treatment, centrifuged (5 min, 300*g*) and resuspended in Hibernate-E medium (Gibco) supplemented with B27 (Invitrogen) and Pen-Strep (10 U ml^−1^ penicillin and 100 µg ml^−1^ streptomycin, Invitrogen). Cell suspensions were stored at 4°C in the dark during overnight shipment. Neurons were plated in Neurobasal medium with B27 and Pen-Strep on a poly-l-lysine (Invitrogen) coating within 24–30 h of dissociation until 2–3 d *in vitro*. Grids were blotted on both sides for 8–10 s and plunge-frozen in liquid ethane using a Vitrobot (FEI) set at 37°C and 80% humidity.

### 3.2. Cryo-electron tomography data collection of cultured neurons   

Single-axis cryo-electron tomography of vitrified neurons was performed using a Tecnai G2 Polara at 300 kV with a Quantum post-column energy filter (Gatan) operated in zero-loss imaging mode with a 20 eV energy-selecting slit. Data at 5–6 µm defocus were collected on a K2 Summit direct electron detector (Gatan) operating in counting mode with a final sampling of 5.39 Å per pixel at 5 e^−^ per pixel per second. Tilt series of ∼110 e^−^ Å^−2^ total dose were collected in 3° increments using the Hagen dose-symmetric tilt scheme (Hagen *et al.*, 2017 ▸) to maxima of −60 and 60°. For each tilt, movies at three or four subframes per second were aligned using *MotionCor*2 (Zheng *et al.*, 2017 ▸). Tomograms from seven neurons were collected and analysed, with tomograms from three neurons being quantitatively assessed in detail.

### 3.3. Cryo-electron tomography image processing and analysis of MTs in cells   

Owing to challenges in achieving useful gold fiducial distribution during sample preparation, fiducial-less alignment on each tilt series was performed *via* patch tracking in the Etomo graphical user interface to *IMOD* (v.4.9.0). CTF determination on each aligned tilt series was performed with *CTFFIND*4 (Rohou & Grigorieff, 2015 ▸) and three-dimensional CTF correction and tomogram reconstruction was performed by weighted back-projection of dose-compensated tilt series with *novaCTF* (Turoňová *et al.*, 2017 ▸). Dose compensation of tilt series was performed using modified custom scripts provided by Dr Ben Bammes (Wang *et al.*, 2014 ▸).

### 3.4. Ultrastructure of MTs in neurons   

**Figure 6:**
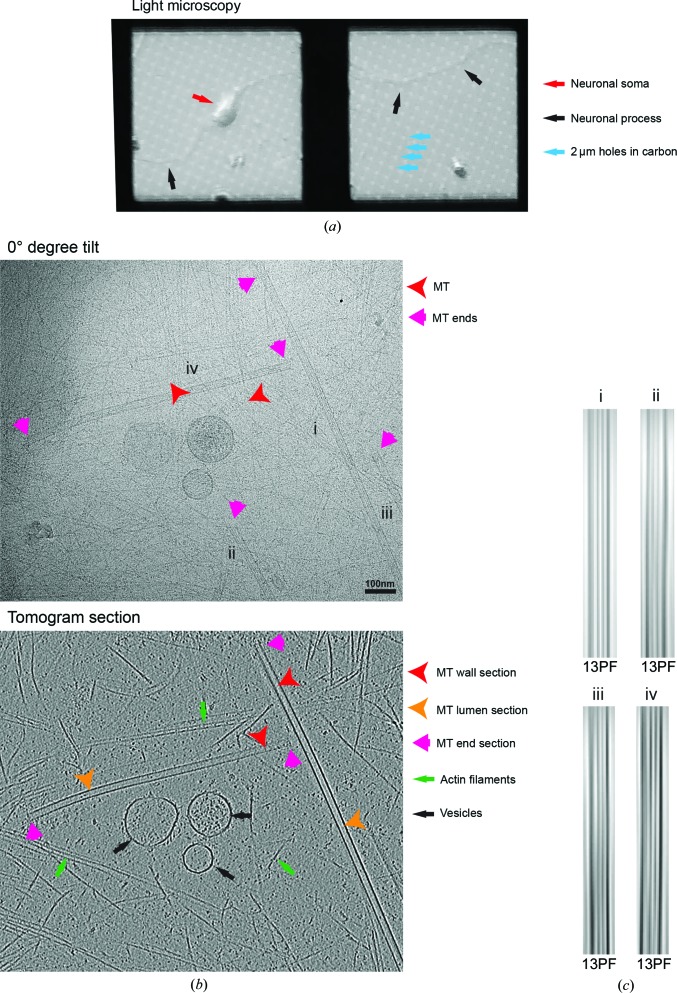
Characterization of MTs in cultured mouse neurons. (*a*) Phase-contrast light-microscope overview of mouse cortical neurons growing on cryo-EM grids. (*b*) Top, 0° tilt image (motion-corrected movie sum; 2.7 e^−^ Å^−1^ total dose) showing a view of the periphery of a neuron lying across a hole in the cryo-EM grid carbon layer, in which numerous cellular components, including MTs (four are labelled i–iv), are visible. Bottom, 8 nm section through the three-dimensional tomographic reconstruction of the same sample, showing the appearance of sections through the MT wall (red arrowheads), the MT lumen (orange arrowheads), MT ends (pink arrows), actin filaments (green arrows) and membranous organelles (black arrows). (*c*) Top, Fourier filtered images of the selected four MTs in (*a*) are shown, which reveal the unvarying moiré pattern typical of 13PF MTs seen in all neuronal MTs examined (*n* = 61). For a comparison with the PF distribution of *in vitro*-polymerized mouse brain tubulin, see Vemu *et al.* (2017 ▸).

The low-magnification light-microscopy view of the cultured neurons grown on a cryo-EM grid (Fig. 6 ▸ *a*) shows the scale and diversity of cellular morphology compared with the 2 µm holes in the carbon film over which the cells appear to readily grow. The task of characterizing the many facets of the MT cytoskeleton in these cells is large, and we summarize just a few preliminary observations here. The majority of the cell soma is too thick (>0.5 µm) to vitrify when using conventional plunge-freezing or to allow sufficient electron transmission during imaging without sectioning. However, regions at the cell periphery, including parts of neuronal processes, are well preserved and show a wealth of detail both in projection and in tomographic sections (Fig. 6 ▸ *b*). MTs are readily identified, often surrounded by actin filaments and interspersed with organelles and vesicles.

Approaches for studying MTs *in vitro* can be applied to these more complex data and inform their interpretation. Evaluation of the MT moiré pattern shows that these MTs are all built from 13 PFs (Fig. 6 ▸ *c*; *n* = 61), in contrast to the range of MT architectures seen for dynamic MTs *in vitro* made of both bovine (Fig. 4 ▸ *b*) and mouse brain tubulin (Vemu *et al.*, 2017 ▸). Often both ends of the MTs are visible. Because these peripheral regions are typically located tens of micrometres away from the centrosome in the cell soma, and because in any case the neuronal centrosome loses its MT-nucleating ability through development (Stiess *et al.*, 2010 ▸), alternative mechanisms to centrosome-mediated nucleation must be in place to preserve this 13PF architecture. This includes *de novo* MT nucleation potentially regulated by MAPs such as EBs (Vitre *et al.*, 2008 ▸) and doublecortin (Moores *et al.*, 2004 ▸), and/or the severance and release of more centrally nucleated MTs followed by their transport into cellular processes. These short MTs are presumably stabilized during transport, and this may involve CAMSAP2, which localizes to noncentrosomal neuronal MTs and is required for neuronal polarization (Chuang *et al.*, 2014 ▸; Yau *et al.*, 2014 ▸). Presumably mechanisms also exist to control the polarity of these peripheral MTs (see, for example, Tas *et al.*, 2017 ▸), and our future work will be directed at further investigating this interesting problem.

**Figure 7:**
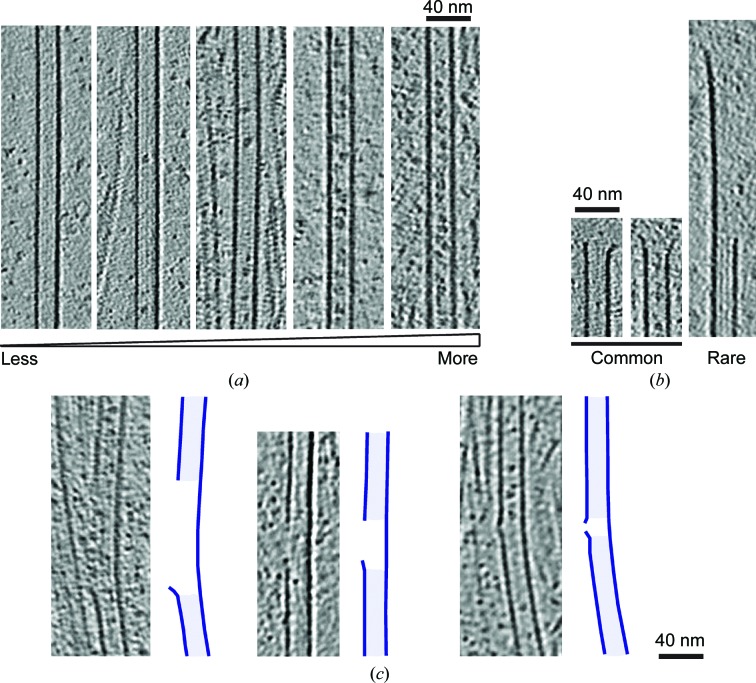
Diversity of MT ultrastructure in neurons. (*a*) A range of particle sizes and distributions are seen in the lumen of neuronal MTs. (*b*) The majority (24/25) of neuronal MT ends have relatively short flared regions similar to those observed in GMPCPP-stabilized MTs (Fig. 2 ▸ *a*), while only one example of a longer curved sheet extension was seen and no highly curved ends associated with *in vitro* depolymerizing MTs (Fig. 5 ▸ *a*) were observed. (*c*) Three examples of lattice defects observed in neuronal MTs. In each case, a section through the tomogram is depicted on the left and a surface rendering of the MT wall is shown on the right.

Our initial characterization of neuronal MTs reveals some further interesting parallels with *in vitro*-polymerized MTs. These MTs presumably have neuronal MT-associated proteins (MAPs) bound to them, but there is little evidence of regular decoration and the MT walls are relatively smooth. Whereas dynamic MTs *in vitro* may accumulate unpolymerized tubulin in their lumen (Fig. 4 ▸ *a*), sections through MTs in neurons reveal particles with a range of sizes and densities (Fig. 7 ▸ *a*), as described previously (Garvalov *et al.*, 2006 ▸). The density of lumenal particles can be very different even in MTs that lie adjacent to each other, and could be determined in part by the density of cellular contents that were present as the MT polymerized; if this was elsewhere within the cell followed by transport into the periphery, the contents of its lumen could reflect that. The identities of these particles are currently unknown; however, as well as cellular contents accumulated by chance, these particles could include tubulin acetylases (Szyk *et al.*, 2014 ▸) since the primary site of tubulin acetylation is in the MT lumen.

Most of the MT ends that we observed in neurons (Fig. 7 ▸ *b*; *n* = 24) displayed a morphology similar to that observed for GMPCPP MTs *in vitro* (Fig. 2 ▸ *a*), *i.e.* with a relatively short curved sheet region. Only a single example of a more extended sheet at the MT end was observed, and no examples of the classical ‘rams horn’ appearance of depolymerizing MT ends were seen. This could be because no depolymerizing MTs were captured in our neurons, perhaps because of the presence of regulatory MAPs, or because the morphology of depolymerizing MTs in neurons involves less pronounced curvature of PFs at their ends.

Finally, and strikingly, lattice defects of various sizes were also observed in neuronal MTs (Fig. 7 ▸ *c*), consistent with the idea that the dynamics and properties of cellular MTs may be subject to regulation within the lattice as well as at their ends (Aumeier *et al.*, 2016 ▸; Dimitrov *et al.*, 2008 ▸; Srayko *et al.*, 2006 ▸). Lattice defects in cells have been speculated to arise from a range of mechanisms, including by the activity of MT-severing enzymes and physical interactions in the crowded cellular context. Lattice defects may act as substrates for regulatory MAPs (Davis *et al.*, 2002 ▸) and influence stepping by transport motors (Liang *et al.*, 2016 ▸). While the origins of these defects *in vitro* and in cells are clearly different, the ability to study these discontinuities *in vitro* will allow understanding of their properties and impact in cells.

## 4. Conclusion and future prospects   

The technologies leading to the ‘resolution revolution’ (Kühlbrandt, 2014 ▸) have dramatically increased the information content of all cryo-EM data, shedding light on numerous dynamic cellular processes. Here, we have highlighted the power of cryo-electron tomography in studying the diversity of MT ultrastructure, thereby shedding light on the molecular mechanisms of dynamic instability. MTs polymerized *in vitro* from solutions of pure tubulin, although experimentally extremely simple compared with cellular MTs, allow the heterogeneity of MT structures to be manipulated and probed in detail. Our data emphasize that several features of these *in vitro*-polymerized MTs, the relatively short sheet-like ends of stable GMPCPP-MTs and the presence of lattice defects, are also found in neuronal MTs. Obviously, the structures of cellular MTs are highly context-dependent, being governed by the specific proteome of a given cell, post-translational modifications of both tubulin and its regulators, and the developmental and physiological status of the cell. As more precise correlated light- and electron-microscopy imaging is achieved, regulators associated with different MT morphologies in cells will be defined (Kukulski *et al.*, 2011 ▸; Wolff *et al.*, 2016 ▸).

*In vitro* reconstitution experiments continue to shed light on the binding preferences of different MAPs. Our study of CAMSAP MT minus-end recognition (Atherton *et al.*, 2017 ▸) highlighted the subtle mechanisms employed by MAPs to recognize the different tubulin conformations associated with regions of the MT cytoskeleton, only a few of which have currently been captured at high resolution (Nogales & Kellogg, 2017 ▸). CAMSAPs are by no means the only MAPs that display such conformational sensitivities: end-binding (EB) proteins specifically recognize growing MT ends and are sensitive to the nucleotide content of the MT lattice (Maurer *et al.*, 2011 ▸) but also bind nonlattice regions of extended MT ends (Guesdon *et al.*, 2016 ▸), while subtle differences in the TOG-domain structures of the XMAP215 and CLASP families of MT regulators may also display sensitivities to different extents of tubulin curvature (Byrnes & Slep, 2017 ▸). Many MAPs show some affinity for tubulin dimers, tubulin oligomers and/or the MT lattice independently of their specific conformation, so it remains a challenge to biochemically differentiate binding mechanisms. The power of cryo-ET to illuminate the range of potential tubulin conformations within a single MT will help to clarify the mechanisms that regulate the diversity of MT function.
